# Clinical and Transcriptomic Characteristics of Aortic Stenosis in Patients Undergoing Haemodialysis

**DOI:** 10.1093/icvts/ivag008

**Published:** 2026-01-08

**Authors:** Satoru Shiraiwa, Nguyen Quoc Vuong Tran, Yosuke Watanabe, Tsuyoshi Kobayashi, Kazuto Nakamura, Chie Nakamura, Soshi Yamamoto, Daichi Shikata, Yuki Takesue, Yoshihiro Honda, Kenji Sakakibara, Shigeaki Kaga, Hiroshi Yokomichi, Atsuhito Nakao, Akira Sato, Hiroyuki Nakajima

**Affiliations:** Department of Surgery II, University of Yamanashi, Yamanashi, Chuo, Yamanashi 409-3898, Japan; Department of Immunology, University of Yamanashi, Chuo, Yamanashi 409-3898, Japan; Department of Cardiology, Faculty of Medicine, University of Yamanashi, Chuo, Yamanashi 409-3898, Japan; Department of Cardiology, Faculty of Medicine, University of Yamanashi, Chuo, Yamanashi 409-3898, Japan; Department of Cardiology, Faculty of Medicine, University of Yamanashi, Chuo, Yamanashi 409-3898, Japan; Department of Surgery II, University of Yamanashi, Yamanashi, Chuo, Yamanashi 409-3898, Japan; Department of Surgery II, University of Yamanashi, Yamanashi, Chuo, Yamanashi 409-3898, Japan; Department of Surgery II, University of Yamanashi, Yamanashi, Chuo, Yamanashi 409-3898, Japan; Department of Surgery II, University of Yamanashi, Yamanashi, Chuo, Yamanashi 409-3898, Japan; Department of Surgery II, University of Yamanashi, Yamanashi, Chuo, Yamanashi 409-3898, Japan; Department of Surgery II, University of Yamanashi, Yamanashi, Chuo, Yamanashi 409-3898, Japan; Department of Surgery II, University of Yamanashi, Yamanashi, Chuo, Yamanashi 409-3898, Japan; Department of Epidemiology and Environmental Medicine, University of Yamanashi, Chuo, Yamanashi 409-3898, Japan; Department of Immunology, University of Yamanashi, Chuo, Yamanashi 409-3898, Japan; Department of Cardiology, Faculty of Medicine, University of Yamanashi, Chuo, Yamanashi 409-3898, Japan; Department of Surgery II, University of Yamanashi, Yamanashi, Chuo, Yamanashi 409-3898, Japan

**Keywords:** aortic stenosis, heamodialysis, RNA sequencing, valve calcification, immune response

## Abstract

**Objectives:**

Patients with aortic stenosis (AS) undergoing haemodialysis (HD) often experience more rapid disease progression and poorer prognosis than non-dialysis patients; however, the underlying mechanisms remain unclear. This study aimed to elucidate clinical and molecular differences between HD and non-HD patients with AS, focusing on transcriptomic profiling of resected aortic valves.

**Methods:**

We retrospectively analysed 183 patients with severe AS who underwent surgical aortic valve replacement at the University of Yamanashi Hospital from February 2015 to May 2024. Among them, 34 patients were receiving maintenance HD, while 149 were not. Clinical data, echocardiographic findings, and CT-based valve calcification were assessed. RNA sequencing was conducted on aortic valve specimens from 5 HD and 4 non-HD patients. Differentially expressed genes were identified, followed by enrichment analysis and immune cell profiling using CIBERSORTx.

**Results:**

Haemodialysis patients exhibited lower body mass index, a higher prevalence of ischaemic heart disease, elevated C-reactive protein and B-type natriuretic peptide levels, and impaired diastolic function compared to non-HD patients. RNA sequencing revealed 35 upregulated and 30 downregulated genes in HD valves. Enrichment analysis demonstrated that genes involved in immune response and ossification were upregulated in aortic valves from HD patients. CIBERSORTx analysis suggested increased macrophage infiltration. Comparison with public datasets identified HD- associated gene signatures.

**Conclusions:**

Patients with AS on HD exhibited distinct clinical features and gene expression profiles. Upregulation of immune and ossification-related genes, alongside macrophage infiltration, suggests a key role for immune response in AS progression among HD patients.

## Graphical abstract

**Figure:**
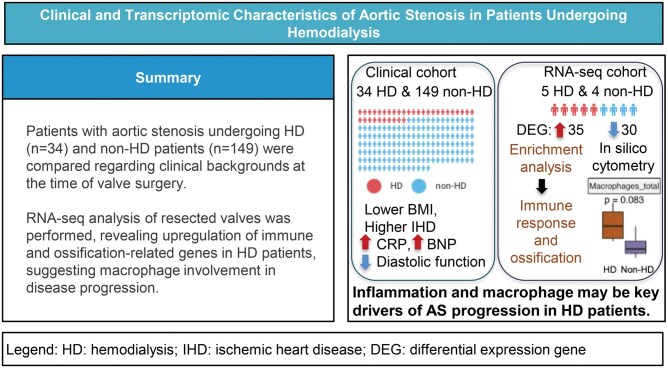

## INTRODUCTION

Chronic kidney disease (CKD) is a major global public health concern, with an estimated global prevalence of 9.1%.^1^ Moreover, it is estimated that around 3.9 million individuals were receiving kidney replacement therapy (including heamodialysis [HD]) for end-stage kidney disease.^2^ CKD is a well-established risk factor for cardiovascular disease and is particularly associated with the progression of aortic stenosis (AS). The rate of AS progression increases as renal function declines, and in patients receiving HD, the progression of AS is approximately 2-fold faster than in non-dialysis patients.^3^ In those with pre-existing valvular calcification, the heamodynamic deterioration of AS progresses even more rapidly.^4^ In patients undergoing HD, aortic valve calcification is clinically known to be mainly associated with phosphate retention-induced secondary hyperparathyroidism and the subsequent development of hypercalcemia.^5^ Pathobiologically, oxidative stress and inflammation have also been implicated in the pathogenesis of calcific aortic valve disease, although the precise mechanisms remain incompletely understood.^6^ Comprehensive transcriptomic data obtained from RNA sequencing of human tissues have profoundly advanced our understanding of disease mechanisms across a wide range of pathological conditions.^7^ Although several transcriptomic studies have analysed human aortic valve tissues using RNA sequencing,^8^ no investigations to date have examined aortic valve specimens obtained from patients undergoing HD.

In this study, we compared the clinical characteristics of HD and non-HD patients with AS who underwent surgical aortic valve replacement (SAVR). Furthermore, we performed RNA sequencing on the excised aortic valves to analyse differences in gene expression profiles between the 2 groups.

Although pilot-scale analysis, our findings suggest that gene groups related to immune responses and calcification may be involved in the development and progression of AS in HD patients.

## METHODS

### Ethics statement

This study complies with the principles of the Declaration of Helsinki and the WMA Declaration of Taipei. The study, including the use of the database generated from the obtained RNA sequencing data, was approved by the Ethics Committee of the University of Yamanashi Hospital (approval number: 2546, approval date: January 7, 2022). Written informed consent was obtained from all patients prior to RNA sequencing analysis of resected aortic valve tissues.

### Study design

Patients who underwent SAVR between February 2015 and May 2024 at the University of Yamanashi Hospital were retrospectively studied. The decision regarding surgical indication was made following discussion by the heart team, in accordance with the Guidelines for Surgical and Interventional Treatment of Valvular Heart Disease issued by the Japanese Circulation Society in 2012*,* and the updated JCS/JSCS/JATS/JSVS 2020 Guidelines on the Management of Valvular Heart Disease.^9^ Among the included patients, 34 were receiving regular haemodialysis (HD group), while 149 were not (non-HD group).

### Patient characteristics and assessment

Patient characteristics included preoperative comorbidities, smoking history, echocardiographic findings, laboratory data, and antihypertensive medication use. Arteriosclerosis indicators, such as the Ankle-Brachial Index (ABI) and pulse wave velocity (PWV), were measured. The level of aortic valve calcification was evaluated by calculating the Agatston score from computed tomography images using SYNAPSE VINCENT software (Fujifilm Corporation, Tokyo, Japan).

### RNA sequencing

Aortic valve tissues resected from patients who underwent SAVR after January 17, 2022, were analysed. Consecutive cases were enrolled, including 5 HD and 4 non-HD patients. The excised aortic valve tissues were promptly frozen in liquid nitrogen and preserved at −80 °C until further analysis. The aortic valves were pulverized under frozen conditions, and total RNA was extracted using the RNeasy Mini Kit (Qiagen, Tokyo, Japan) in accordance with the manufacturer’s protocol. RNA sequencing was performed by Macrogen Japan (Tokyo, Japan) using the NovaSeq 6000 platform (2 × 150 bp configuration). Poly(A) selection was used for mRNA enrichment, and raw FASTQ data were obtained for downstream gene expression analysis. Quality control and adapter trimming were performed on raw FASTQ files. Reads were aligned to the reference human genome (hg38). Raw counts from aligned reads were used for differential gene expression analysis with DESeq2.

### Differential gene expression analysis

Differential gene expression analysis was performed using DESeq2 to identify differentially expressed genes (DEGs) between AS patients without and with HD.^10^ Raw read counts from RNA sequencing (RNA-seq) were extracted and pre-processed to remove lowly expressed genes. The counts matrix was then imported into DESeq2. By default, DESeq2 applies the Benjamini-Hochberg procedure to control the false discovery rate.

### Enrichment analysis

Following DEG identification, functional enrichment analysis was performed on significant DEGs to interpret the biological pathways and processes involved. Gene Ontology (GO) analysis was conducted using the R package clusterProfiler.^11^

### Pathway analysis

Pathway analysis was conducted using the refined Hallmark pathways provided by the Molecular Signatures Database.^12^^,^^13^ The Hallmark pathways were utilized to reduce redundancy.^12^ We then performed ANOVA and applied the Benjamini-Hochberg correction method to compare the enrichment score of each pathway between HD and non-HD AS patients and to minimize false-positive results.

### Interaction network analysis using STRING

Protein-protein interaction (PPI) analysis of upregulated and downregulated DEGs was conducted using STRING (version 12.0) (https://string-db.org/) with default parameters.^14^ The PPI enrichment *P*-value, extracted from the analysis tab, indicates whether the input list exhibits more interactions than would be expected from a random list of the same size.

### Immune cell proportion analysis with CIBERSORTx

The abundance of immune cells in aortic valve samples was estimated using in silico cytometry via CIBERSORTx.^15^ CIBERSORTx is a machine learning–based method that predicts the abundance of specific cell types from bulk transcriptome data. An expression matrix was generated from the normalized count table, formatted according to platform requirements. The online CIBERSORTx platform (available at https://cibersortx.stanford.edu) with the default panel of 22 immune cell types was used to impute cell fractions for each dataset.

### Statistical analysis

Comparisons of continuous variables were performed using the Mann-Whitney *U* test. Categorical variables were analysed using Fisher’s exact test. A 2-tailed *P*-value <.05 was considered statistically significant. Missing patient data were excluded from the analysis. All statistical analyses were performed using R version 4.4.2 (R Foundation for Statistical Computing, Vienna, Austria) with the EZR package.

## RESULTS

### Patient characteristics

The study included 183 patients who underwent aortic valve replacement due to AS, with 34 patients in the HD group and 149 in the non-HD group. The characteristics of AS patients with and without HD are summarized in **Table 1**. The causes of dialysis were as follows: diabetic nephropathy (47.1%), chronic glomerulonephritis (38.2%), focal segmental glomerulosclerosis (5.9%), gouty nephropathy (2.9%), polycystic kidney disease (2.9%), and trauma (2.9%). Among the factors other than HD that influence AS, the bicuspid aortic valve was significantly more frequent in the non-HD patients. Compared with non-HD patients, HD patients had a lower body mass index and a higher prevalence of ischaemic heart disease. There were no significant differences in age, sex distribution, smoking status, or history of cerebrovascular disease between the 2 groups. Echocardiographic findings revealed no significant differences between HD and non-HD patients in peak aortic jet velocity, left ventricular ejection fraction. There were no significant differences in ABI between HD and non-HD patients; however, PWV was significantly higher in HD patients. Similarly, no significant differences were observed in aortic valve calcification between the 2 groups. Both the calcification volume and the Agatston score were comparable. Compared to non-HD patients, HD patients exhibited lower levels of haemoglobin, platelet count, and albumin, while showing elevated levels of C-reactive protein (CRP), phosphate, and B-type natriuretic peptide (BNP).

**Table 1.** Characteristics of Study Patients

	Total	HD	Non-HD	*P*-value
	(*n* = 183)	(*n* = 34)	(*n* = 149)	
Age, (years)	75 [69-80]	7 [68-77]	75 [70-80]	.07
Male sex	113 (61.7%)	23 (67.6%)	90 (60.4%)	.56
BMI (kg/m^2^)	22.1 [20.5-25.1]	20.5 [19.6-21.6]	22.7 [20.9-25.4]	<.01
Smoking	71 (38.8%)	11 (32.4%)	60 (40.3%)	.44
Diabetic nephropathy	25 (13.7%)	16 (47.1%)	9 (6%)	<.01
CGN	13 (7.1%)	13 (38.2%)	–	
FGS	2 (1.1%)	2 (5.9%)	–	
Gouty nephropathy	1 (0.5%)	1 (2.9%)	–	
PKD	1 (0.5%)	1 (2.9%)	–	
Trauma	1 (0.5%)	1 (2.9%)	–	
History of IHD	54 (29.5%)	17 (50%)	37 (24.8%)	<.01
Autoimmune disease	5 (2.7%)	1 (2.9%)	4 (2.7%)	1.00
Systemic inflammatory disease	1 (0.5%)	0 (0%)	1 (0.7%)	1.00
Bicuspid aortic valve	39 (21.3%)	1 (2.9%)	38 (25.5%)	<.01
EF (%)	65 [53-73]	62 [50-69]	66 [56-73]	.06
AV peak V (m/s)	4.4 [3.8-4.8]	4.3 [3.8-4.6]	4.4 [3.8-4.8]	.50
Hb (g/dL)	12.3 [11.1-13.7]	11.0 [10.1-11.4]	12.7 [11.3-13.9]	<.01
WBC (×10^3^/μL)	5.3 [4.4-6.4]	5.1 [3.9-6.3]	5.3 [4.4-6.4]	.21
Platelet (×10^3^/μL)	180 [141-210]	158 [128-183]	186 [150-215]	<.01
Albumin (g/dL)	4 [3.7-4.2]	3.5 [3.1-3.7]	4.1 [3.8-4.3]	<.01
CRP (mg/dL)	0.1 [0.1-0.2]	0.2 [0.1-1.0]	0.1 [0.1-0.2]	<.01
Creatinine (mg/dL)	1.0 [0.8-1.5]	7.2 [6.4-8.3]	0.9 [0.7-1.2]	<.01
eGFR (mL/min/1.73m^2^)	49 [32-69]	6 [5-7]	56 [43-71]	<.01
CKD stage				
G1	7(3.8%)	0 (0%)	7 (4.7%)	
G2	60(32.8%)	0 (0%)	60 (40.3%)	
G3a	41(22.4%)	0 (0%)	41 (27.5)	
G3b	34(18.6%)	0 (0%)	34 (40.3%)	
G4	7(3.8%)	0 (0%)	7 (4.7%)	
G5	34(18.6%)	34 (0%)	0 (0%)	
Calcium (mg/dL)	9.3 [8.9-9.5]	9.0 [8.4-9.4]	9.3 [9-9.5]	.03
Phosphorus (mg/dL)	3.7 [3.4-4.2]	5.0 [4.1-5.9]	3.5 [3.2-4.0]	<.01
BNP (pg/mL)	248 (87-613)	1231 (616-2387)	202 (74-403)	<.01
mABI	1.1 [1.0-1.1]	1.0 [0.9-1.2]	1.1 [1.0-1.1]	.30
mPWV (cm/s)	1391 [1226-1692]	1914 [1481-2250]	1357 [1208-1562]	<.01
Calcification volume (mm^3^)	2544 [1482-4037]	2848 [1701-4149]	2450 [1410-4001]	.43
Agaston score	1951 [1069-3216]	2147 [1205-3154]	1928 [1051-3222]	.67

Data are expressed as the median and range (25th and 75th percentiles) or number (%). *P* values, comparison between HD and non-HD patients.

Abbreviations: AV Peak V, aortic valve peak velocity; BMI, body mass index; BNP, B-type natriuretic peptide; CGN, chronic glomerulonephritis; CKD, chronic kidney disease; CRP, C-reactive protein; EF, ejection fraction; FGS, Focal Segmental Glomerulosclerosise; eGFR, estimated glomerular filtration rate; Hb, hemoglobin; IHD, ischaemic heart disease; mABI, mean ankle-brachial index; mPWV, mean pulse wave velocity; PKD: polycystic kidney disease; WBC, white blood cell.

### Differential expressed genes and enrichment analyses

**Figure 1.**
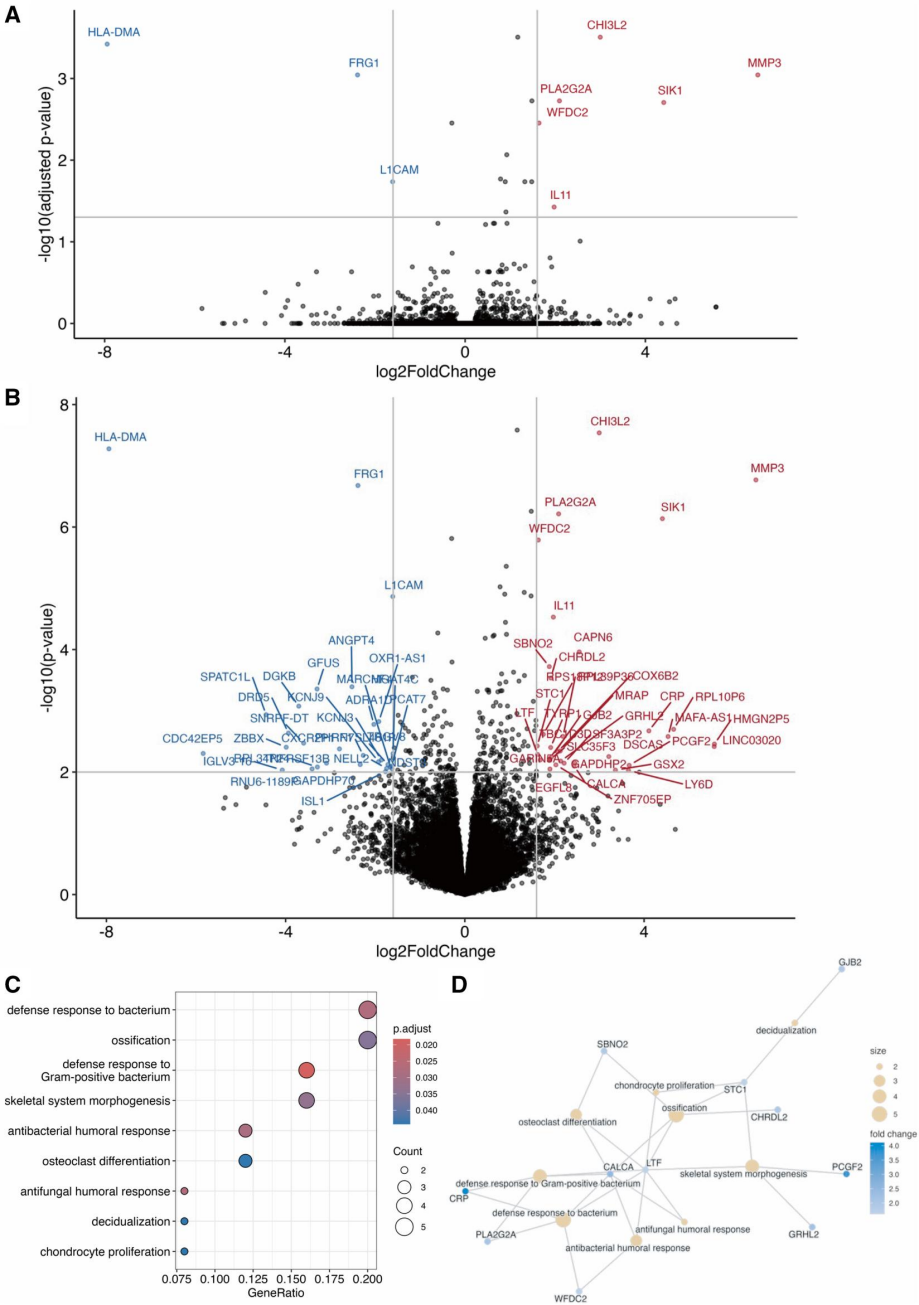
DGE Analysis Between Non-HD and HD Patients. (A) Volcano plot showing significantly differentially expressed genes (adjusted *P*-value <.05 and |log_2_FoldChange| > 1.6) between non-HD and HD patients with aortic stenosis. Red points represent upregulated genes (*n* = 6), and blue points represent downregulated genes (*n* = 3). (B) Volcano plot showing differentially expressed genes used for exploratory investigation (*P*-value <.01 and |log_2_FoldChange| > 1.6) between non-HD and HD patients with aortic stenosis. Red points represent upregulated genes (*n* = 35), and blue points represent downregulated genes (*n* = 30). (C) Dot plot showing GO term enrichment analysis of 35 upregulated genes in the exploratory investigation. Dot colour indicates adjusted *P*-value (as shown in the colour scale), and dot size represents the number of genes associated with each term. (D) Functional network of genes enriched in significant GO terms. Nodes (grey circles) represent individual genes, and edges indicate functional interactions or shared biological processes. Abbreviations: GO, Gene Ontology; HD, haemodialysis.

To gain insights into the differences between aortic valves from HD and non-HD AS patients, we performed RNA sequencing followed by DEG analysis using surgically resected aortic valves from 4 non-HD AS and 5 HD patients. **Table 2** presents individual patient data, whereas **Table S1** summarizes the results. Due to the small sample size and limited number of significantly DEGs when applying the adjusted *P* value <.05 (6 and 3 genes were significantly upregulated and downregulated in HD patients, respectively; **Figure 1A**), we considered genes with *P*-value <.01 and absolute fold change >3 (ie, |log_2_FoldChange| > 1.6) as significantly differentially expressed for an exploratory investigation. Based on this criterion, 35 genes were upregulated and 30 were downregulated in the aortic valves of HD patients (**Figure 1B**; **Tables S2 and S3**).

**Table 2.** Characteristics of Patients in RNA-Sequencing

Patient	Age	Sex	BMI	AV Peak V	EF	Hb	WBC	PLT	Alb	CRP	CRE	CKD	Ca	P	BNP	mPWV	Ca volume	Agaston
			kg/m^2^	m/s	%	g/dL	×10^3^/μL	×10^3^/μL	g/dL	mg/dL	mg/dL	stage	mg/dL	mg/dL	pg/mL	cm/s	mm^3^	score
HD①	80	M	19.63	4.4	69.5	11.7	6.89	183	3.1	1.18	4.15	G5	8.6	3.5	174	1843	3156	2486
HD②	57	M	23.86	4.7	40.3	10.3	7.57	299	3.6	0.23	7.37	G5	9.3	4.2	1953	2070	8790	6828
HD③	72	F	19.67	4.5	49.4	13.7	4.58	196	3.5	0.18	6.94	G5	—	—	1374	1961	1336	980
HD④	72	F	20.65	4.5	77.8	11.2	5.52	182	4.1	0.1	5.07	G5	8.9	4.2	271	1898	1314	990
HD⑤	56	F	27.00	4.3	64	11.8	5.73	181	3.7	0.27	6.58	G5	9.7	6.6	223	—	1450	917
Non-HD①	74	F	22.38	4.9	45.2	12.3	5.66	162	4	0.12	0.72	G3a	9.2	3.8	309	1041	3447	2690
Non-HD②	77	M	25.52	4.28	79.2	14.5	6.87	196	4.2	0.12	1.31	G3b	9.9	4.3	422	1823	4241	3254
Non-HD③	57	F	28.95	5.1	71.5	15.2	8.61	111	4.6	0.14	0.68	G2	10	4.6	35	1047	1237	975
Non-HD④	74	F	32.28	3.8	59.8	11.9	6.58	215	3.9	0.1	0.73	G3a	9.6	4.2	34	1541	2871	1947

Abbreviations: AV Peak V, aortic valve peak velocity; BMI, body mass index; BNP, B-type natriuretic peptide; Ca, calcium; CKD, chronic kidney disease; CRE, creatinine; CRP, C-reactive protein; EF, ejection fraction; Hb, hemoglobin; HD, haemodialysis; mPWV, mean pulse wave velocity; P, phosphorus; WBC, white blood cell.

Due to the low number of significantly DEG lead to no significant GO term for genes in **Figure 1A**. In an exploratory investigation, GO enrichment analysis of the upregulated genes revealed enrichment in 2 major categories: immune responses (eg, defence responses to Gram-positive bacteria; general antibacterial and antifungal humoral responses) and ossification processes (eg, skeletal system morphogenesis, ossification, and osteoclast differentiation). Ten genes were annotated to immune response- and ossification-related pathways, including *CALCA, CRP, LTF, PLA2G2A, WFDC2, GRHL2, PCGF2, STC1, CHRDL2,* and *SBNO2* (**Figure 1C and D**; **Table S4**). No significant enrichment was observed among the downregulated genes.

**Figure 2.**
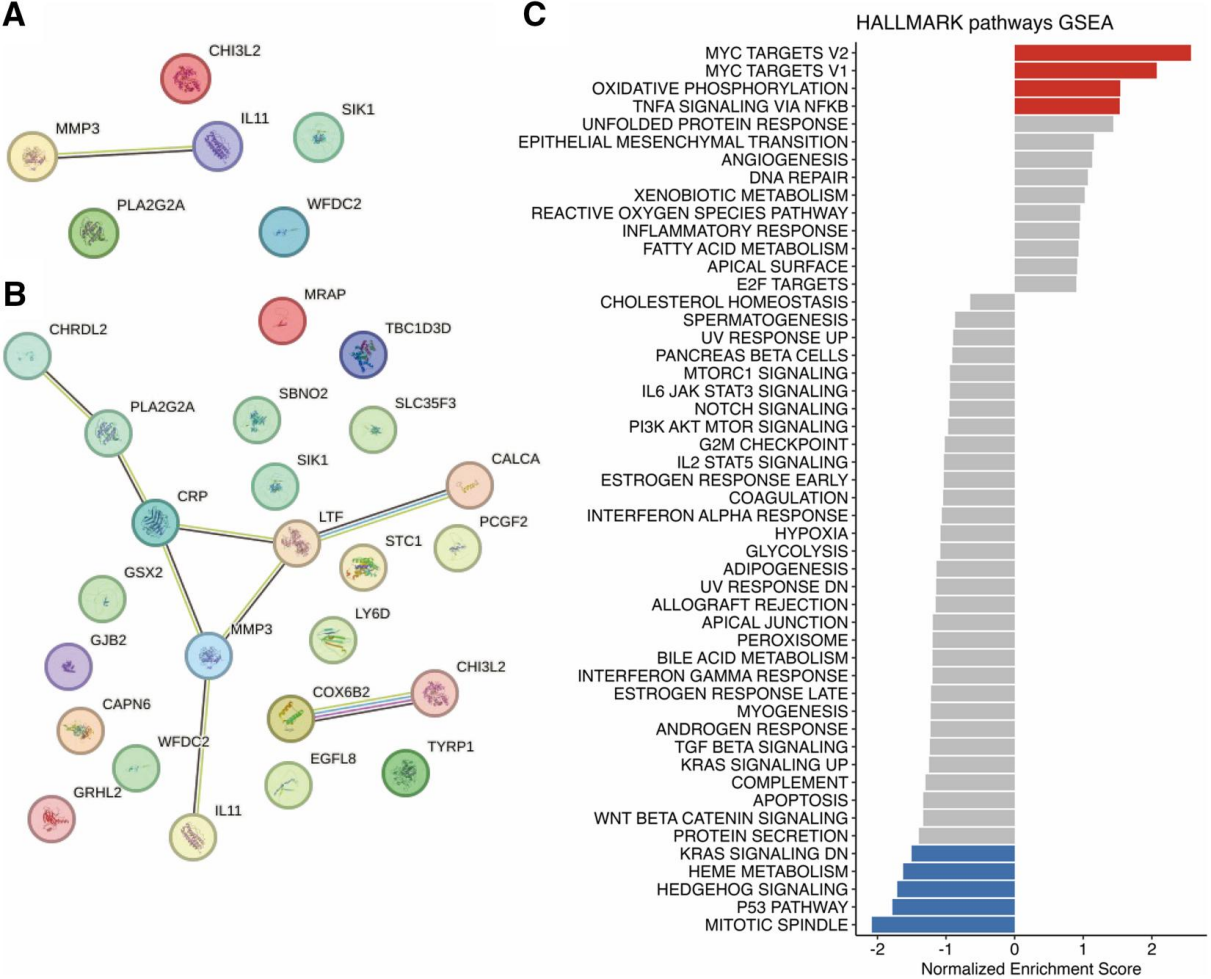
Protein-Protein Interaction (PPI) Network and Pathway Analysis. (A) PPI network for the 6 significantly upregulated genes. No significant interaction was predicted. (B) PPI network for the 35 upregulated genes in the exploratory investigation. A significant interaction axis involving *CALCA, LTF, CRP, PLA2G2A, CHRDL2, MMP3,* and *IL11* was predicted. (C) Pathway analysis of Hallmark pathways conducted using the Gene Set Variation Analysis (GSVA) method to compare differences between non-HD and HD patients. Bars represent the variation in normalized enrichment scores between the groups. Pathways shown in blue indicate statistically significant enrichment differences.

Consistently, PPI network analysis using STRING did not predict any significant interaction involving significantly DEGs while predicting an interaction axis involving *CALCA, LTF, CRP, PLA2G2A, CHRDL2, MMP3,* and *IL11* in the exploratory investigation, supporting their coordinated roles in immune response and calcification in HD valves (**Figure 2A and B**).

### Pathway analysis

To complement the DEG- and GO-based insights, we next performed pathway analysis using the Hallmark gene set collection, which aggregates genes into biologically coherent modules. Whereas GO enrichment is based strictly on the subset of significantly upregulated genes, Hallmark pathway analysis evaluates transcriptome-wide expression patterns and can reveal broader regulatory shifts.

This analysis revealed the higher enrichment of gene sets associated with c-Myc proto-oncogene (MYC) targets V1, MYC targets V2, oxidative phosphorylation, and tumor necrosis factor-α (TNF-α) signalling via nuclear factor-κB (NF-κB) (**Figure 2C**; **Table S5**). These findings suggest that, beyond the localized upregulation of calcification-related genes, ASHD valves are associated with global transcriptional programmes associated with cellular proliferation, metabolic activation, and inflammatory signalling. Taken together, the GO terms highlight specific genes directly involved in calcification and immune response, while the Hallmark pathways reveal systemic activation of stress, immune, and metabolic circuits likely contributing to the overall pathology of calcified valves in HD patients.

### Comparative analysis using public transcriptome data

Although this study did not include normal aortic valve tissue as a control, we utilized previously published transcriptomic data (GSE51472)^8^ comparing normal and stenotic aortic valves to further clarify gene expression changes specific to aortic valves from HD patients. Of the 35 genes upregulated in HD valves, 24 were also represented in GSE51472, allowing us to stratify them into 2 expression profiles.

**Figure 3.**
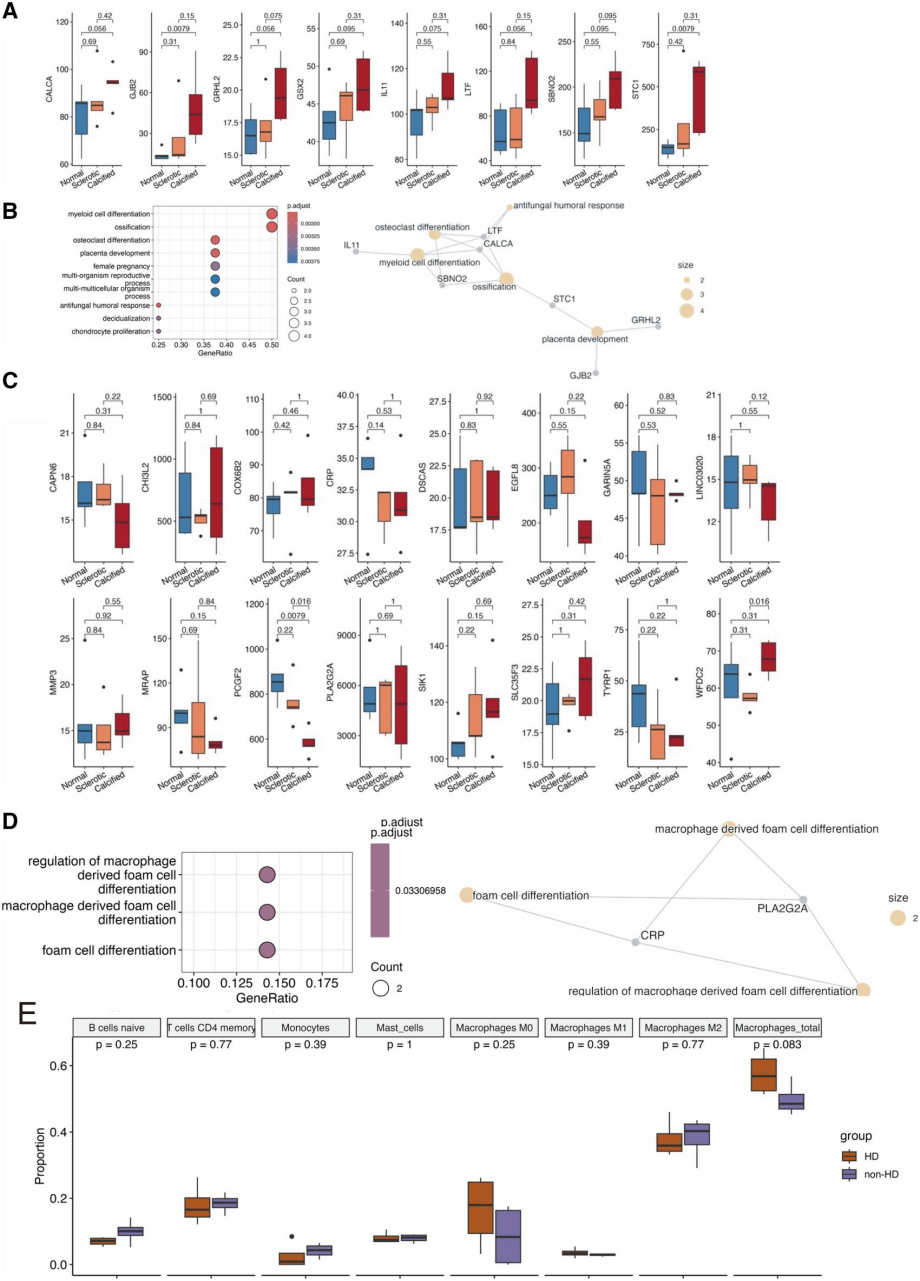
Comparative Transcriptome Analysis With Publicly Available Data. (A) Box plot showing the expression of 8 genes that were upregulated in both sclerotic and calcified aortic valves in GSE51472 and in aortic valves from HD patients in our dataset. *P*-values were calculated using pairwise Wilcoxon tests with correction for multiple testing. (B) Dot plot (left panel) displaying GO term enrichment analysis, and functional gene network (right panel) of the 8 genes shown in panel A. Dot colour indicates the adjusted *P*-value, as shown in the scale of the left panel; dot size represents the number of associated genes. (C) Box plot showing the expression of 16 genes that were upregulated in aortic valves from HD patients in our dataset but not upregulated in sclerotic or calcified valves in GSE51472. *P*-values were calculated using pairwise Wilcoxon tests with correction for multiple testing. (D) Dot plot (left panel) displaying GO term enrichment analysis, and functional gene network (right panel) of the 16 genes shown in panel C. Dot colour indicates the adjusted *P*-value, as shown in the scale of the left panel; dot size represents the number of associated genes. (E) Box plot showing the predicted proportions of selected immune cell types from CIBERSORTx analysis. *P*-values were calculated using the Wilcoxon test. Abbreviations: GO, Gene Ontology; HD, haemodialysis.

The first group consisted of genes that were upregulated in the aortic valves of HD patients and also upregulated in sclerotic or calcified aortic valves in GSE51472, namely *CALCA*, *GJB2*, *GRHL2*, *GSX2*, *IL11*, *LTF*, *SBNO2*, and *STC1* (**Figure 3A**). These genes are likely upregulated during the pathological processes of aortic valve disease. Gene Ontology enrichment analysis of this group identified “myeloid cell differentiation” and “ossification” as the top enriched categories (**Figure 3B**). Notably, several of these genes (*IL11, LTF, CALCA, SBNO2,* and *STC1*) have been previously associated with calcified aortic valves, suggesting that HD patients exhibit an exaggerated calcification phenotype, potentially mediated by inflammatory and myeloid-lineage signalling pathways.

The second group consisted of genes that were upregulated in the aortic valves of HD patients but not upregulated in sclerotic or calcified valves in GSE51472. These included *CAPN6*, *CHI3L2*, *COX6B2*, *CRP*, *DSCAS*, *EGFL8*, *GARIN5A*, *LINC03020*, *MMP3*, *MRAP*, *PCGF2*, *PLA2G2A*, *SIK1*, *SLC35F3*, *TYRP1*, and *WFDC2* (**Figure 3C**). However, GO term enrichment analysis of this group identified only 2 genes—*CRP* and PLA2G2A—which were associated with the term “macrophage-derived foam cell” (**Figure 3D**).

### Digital cytometry analysis using CIBERSORTx

Prompted by the enrichment of immune response—and myeloid-related genes—particularly the involvement of macrophages—we performed in silico cytometry using CIBERSORTx to evaluate immune cell composition in aortic valves. Among the examined immune cell types (see “Materials and Methods”), macrophages, B cells, CD4 memory T cells, monocytes, and mast cells were found to have high proportions in the aortic valves (**Table S6**). We did not observe any significant differences in the proportions of these cell types between non-HD and HD groups (**Figure 3E**), possibly due to the small number of samples included in the analysis. However, an increased trend was observed for the proportion of total macrophages in HD patients (*P* = .083; **Figure 3E**). This trend aligns with the comparative transcriptomic findings and supports the notion that macrophages may contribute to the heightened inflammatory and calcific landscape in HD-associated AS.

## DISCUSSION

This study elucidated the clinical characteristics of dialysis and non-dialysis patients undergoing SAVR and investigated the gene expression profiles of excised aortic valve specimens obtained during surgery.

The clinical features of patients with AS undergoing HD were broadly comparable to those described in previous reports comparing HD with non-HD patients.^16^ Although previous studies have reported that dialysis patients are more prone to progression of aortic valve calcification,^17^^,^^18^ our study demonstrated only a trend towards greater calcification in the dialysis group, without reaching statistical significance. While the Agatston score showed almost no difference, there was a tendency for greater calcium volume in HD patients. The enhanced immune response in the aortic valve suggested by our RNA sequencing results may contribute to more extensive valvular sclerosis in these patients.

Given the small sample size and the inferential nature of deconvolution, these findings are exploratory and require orthogonal validation. Comprehensive transcriptomic analysis using RNA sequencing revealed that a total of 35 genes were significantly upregulated, while 30 genes were significantly downregulated in aortic valve specimens obtained from dialysis patients. Enrichment and PPI analyses suggested that both immune responses and ossification may jointly contribute to the progression of HD-associated AS.

Hallmark pathway analysis, a gene set–based approach that identifies activation of key biological pathways, revealed increased activity of both MYC targets V1 and V2 in HD patients, along with oxidative phosphorylation and TNF-α signalling via NF-κB. Notably, MYC is a master regulatory gene involved in the control of the cell cycle and metabolism.^19^ Although the NF-κB pathway has previously been implicated in the progression of non-HD-associated AS,^20^ our findings suggest that it may also play a significant role in the more severe progression of the disease in HD patients.

Since normal control samples could not be obtained in this study, we compared our results with a previously published transcriptomic dataset (GSE51472)^8^ to investigate whether HD induces unique gene expression changes. Genes that were upregulated in HD and also upregulated in the public dataset—such as *CALCA*, *GJB2*, *GRHL2*, *GSX2*, *IL11*, *LTF*, *SBNO2*, and *STC1*—are considered to be generally associated with conventional AS and may be further enhanced in the setting of HD. In contrast, genes that were upregulated in HD but not in the public dataset—*CAPN6*, *CHI3L2*, *COX6B2*, *CRP, DSCAS*, *EGFL8*, *GARIN5A*, *LINC03020*, *MMP3*, *MRAP*, *PCGF2*, *PLA2G2A*, *SIK1*, *SLC35F3*, *TYRP1*, and *WFDC2*—may represent HD- associated changes that contribute to AS progression.

RNA sequencing has generated numerous clinical discoveries in oncology; however, its application in cardiovascular research remains relatively limited. Nevertheless, RNA sequencing provides an enormous amount of transcriptomic information that can be leveraged to elucidate disease mechanisms. In this study, despite its small sample size, successfully identified several genes that may contribute to AS in patients undergoing HD. Expanding RNA sequencing analyses to larger patient cohorts could further elucidate the molecular mechanisms underlying the progression of AS.

### Study limitations

This study has several limitations. The study is a cross-sectional, single-centre, retrospective design, which also limits causal inference and generalizability, as no external validation was performed (clinical cohort: *n* = 183; RNA-seq cohort: 9 patients). Moreover, RNA sequencing was performed only in a subset of the clinical cohort, and certain characteristics—such as sex distribution—differed between the clinical and RNA-seq cohorts. Bulk tissue analysis may have masked cell type-specific transcriptional signals. In addition, not only HD, but also potential confounding factors, such as comorbidities, phosphate, and calcium levels, may have influenced the results of RNA-seq. Furthermore, the public aortic-valve dataset lacks HD annotation, limiting direct external validation of HD effects. Thus, overlapping/non-overlapping genes in our analysis should not be interpreted as HD specificity. Finally, our study revealed transcriptional associations rather than direct histologic evidence or causal effects. Future studies incorporating immunohistochemistry and functional assays are needed to elucidate the mechanisms by which HD may contribute to AS.

## CONCLUSION

In this study, we characterised the clinical backgrounds of dialysis and non-dialysis patients at the time of SAVR and performed RNA-seq to analyse gene expression profiles in aortic valves from dialysis patients with AS. The RNA-seq results, although a pilot-scale analysis, revealed increased expression of genes related to immune responses and calcification in the aortic valves of HD patients.

## Supplementary Material

ivag008_Supplementary_Data

## Data Availability

The data in this study are available upon request from the corresponding author. The raw RNA-seq FASTQ files and associated metadata have been deposited in the NCBI Gene Expression Omnibus under accession number GSE304300.
