# Supplementary material for: Clinical and Transcriptomic Characteristics of Aortic Stenosis in Patients Undergoing Haemodialysis
Source: Interdiscip Cardiovasc Thorac Surg. 2026 Jan 8;41(1):ivag008. doi: 10.1093/icvts/ivag008 (PMC12861330; doi:10.1093/icvts/ivag008)
Supplement: ivag008_Supplementary_Data [file ivag008_supplementary_data.zip › Table S1 1123.docx]

**TableS1. Characteristics of the RNA-sequencing cohort (n = 9)**

|  | **Total**  **(n=9)** | **HD**  **(n=5)** | **non-HD**  **(n=4)** | **P value** |
| --- | --- | --- | --- | --- |
| **Age, years** | 72[57–74] | 72[57–72] | 74[70–75] | 0.46 |
| **Male sex** | 3(33%) | 2(40%) | 1(25%) | 1.00 |
| **BMI, kg/m^2^** | 24[21–27] | 21[20–24] | 27[25–30] | 0.11 |
| **History of IHD** | 6(66.7%) | 4(80%) | 2(50%) | 0.52 |
| **Autoimmune disease** | 1(11.1%) | 1(20%) | 0(0%) | 1.00 |
| **Systemic inflammatory disease** | 1(11.1%) | 1(20%) | 0(0%) | 1.00 |
| **Bicuspid aortic valve** | 0(0%) | 0(0%) | 0(0%) | 1.00 |
| **EF, %** | 64[49–72] | 64[49–70] | 66[56–73] | 0.71 |
| **AV Peak V, m/s** | 4.5[4.3–4.7] | 4.5[4.4–4.5] | 4.6[4.2–5.0] | 1.00 |
| **Hb, g/dL** | 11.9[11.7–13.7] | 11.7[11.2–11.8] | 13.4[12.2–14.7] | 0.07 |
| **WBC, ×10^3/^μL^3^** | 6.58[5.66–6.89] | 5.73[5.52–6.89] | 6.72[6.35–7.3] | 0.54 |
| **Platelet, ×10^3^/μL^3^** | 183[181–196] | 183[182–196] | 179[149–201] | 0.62 |
| **Albumin, g/dL** | 3.9[3.6–4.1] | 3.6[3.5–3.7] | 4.1[4.0–4.3] | 0.07 |
| **CRP, mg/dL** | 0.14[0.12–0.23] | 0.23[0.18–0.27] | 0.12[0.11–0.12] | 0.14 |
| **Creatinine, mg/dL** | 4.15[0.73–6.58] | 6.58[5.07–6.94] | 0.72[0.71–0.88] | 0.02 |
| **eGFR, mL/min/1.73m²** | 11.6[6.8–58.8] | 6.8[5.8–7.1] | 59.3[54.5–61.9] | <0.01 |
| **Calcium, mg/dL** | 9.5[9.1–9.8] | 9.1[8.83–9.4] | 9.8[9.5–10.0] | 0.19 |
| **Phosphorus, mg/dL** | 4.2[4.1–4.4] | 4.2[4.0–4.8] | 4.3[4.1–4.4] | 0.88 |
| **mPWV** | 6.18[4.32–7.54] | 1929[1871–2016] | 1294[1044–1682] | 0.03 |
| **Calcification volume, mm^3^** | 2871[1336–3447] | 1450[1336–3156] | 3159[2463–3645] | 0.90 |
| **Agaston score** | 1947[980–2690] | 990[980–2486] | 2319[1704–2831] | 0.71 |

Data are expressed as the median and range (25th and 75th percentiles) or number (%). P values, comparison between HD and non-HD patient. AV Peak V：Aortic valve peak velocity, BMI：Body mass index, BNP：B-type natriuretic peptide, CRP：C-reactive protein, EF：Left ventricular ejection fraction, GFR : estimated Glomerular filtration rate, Hb：Hemoglobin, IHD：Ischemic heart disease, mABI：Mean ankle-brachial index, mPWV：Mean pulse wave velocity, WBC：White blood cell
