# Supplementary material for: Clinical and Transcriptomic Characteristics of Aortic Stenosis in Patients Undergoing Haemodialysis
Source: Interdiscip Cardiovasc Thorac Surg. 2026 Jan 8;41(1):ivag008. doi: 10.1093/icvts/ivag008 (PMC12861330; doi:10.1093/icvts/ivag008)
Supplement: ivag008_Supplementary_Data [file ivag008_supplementary_data.zip › Table S3 1121 2.docx]

**Table S3. Significant genes from differential gene expression analysis between HD and non-HD patients**

| gene | baseMean | log2FC | pvalue | padj |  | gene | baseMean | log2FC | pvalue | padj |
| --- | --- | --- | --- | --- | --- | --- | --- | --- | --- | --- |
| **CHI3L2** | 8632.57 | 3.00 | 3.E-08 | 3.E-04 |  | **HLA-DMA** | 77.79 | -7.94 | 5.E-08 | 4.E-04 |
| **MMP3** | 27.38 | 6.50 | 2.E-07 | 9.E-04 |  | **FRG1** | 174.69 | -2.39 | 2.E-07 | 9.E-04 |
| **PLA2G2A** | 13374.04 | 2.09 | 6.E-07 | 2.E-03 |  | **L1CAM** | 1223.69 | -1.61 | 1.E-05 | 0.02 |
| **SIK1** | 34.00 | 4.41 | 7.E-07 | 2.E-03 |  | ANGPT4 | 97.47 | -2.52 | 4.E-04 | 0.23 |
| **WFDC2** | 33.50 | 1.64 | 2.E-06 | 4.E-03 |  | GFUS | 43.99 | -3.30 | 4.E-04 | 0.23 |
| **IL11** | 157.72 | 1.97 | 3.E-05 | 0.04 |  | DGKB | 20.57 | -3.71 | 8.E-04 | 0.33 |
| CAPN6 | 37.77 | 2.55 | 1.E-04 | 0.10 |  | SPATC1L | 14.14 | -4.44 | 1.E-03 | 0.42 |
| SBNO2 | 191.31 | 1.88 | 2.E-04 | 0.16 |  | OXR1-AS1 | 9.30 | -1.92 | 1.E-03 | 0.46 |
| CHRDL2 | 1001.83 | 1.92 | 3.E-04 | 0.20 |  | ADRA1D | 7.43 | -2.03 | 2.E-03 | 0.46 |
| CRP | 5.41 | 4.11 | 2.E-03 | 0.50 |  | DRD5 | 2.79 | -3.94 | 2.E-03 | 0.52 |
| RPL10P6 | 39.52 | 4.66 | 2.E-03 | 0.50 |  | SNRPF-DT | 4.25 | -3.60 | 3.E-03 | 0.61 |
| RPS18P12 | 11.66 | 1.75 | 2.E-03 | 0.54 |  | ZBBX | 2.93 | -3.99 | 4.E-03 | 0.63 |
| MAFA-AS1 | 2.61 | 4.54 | 3.E-03 | 0.54 |  | CXCR2P1 | 23.54 | -2.80 | 4.E-03 | 0.65 |
| RPL39P36 | 7.28 | 2.19 | 3.E-03 | 0.54 |  | CDC42EP5 | 18.89 | -5.84 | 5.E-03 | 0.66 |
| HMGN2P5 | 5.23 | 5.57 | 3.E-03 | 0.63 |  | MGAT4C | 37.64 | -1.60 | 5.E-03 | 0.66 |
| LINC03020 | 5.18 | 5.56 | 4.E-03 | 0.63 |  | PHRF1 | 56.35 | -2.27 | 5.E-03 | 0.67 |
| STC1 | 823.18 | 1.62 | 4.E-03 | 0.63 |  | TRGV8 | 9.10 | -1.60 | 6.E-03 | 0.69 |
| TYRP1 | 24.25 | 1.91 | 4.E-03 | 0.63 |  | KCNJ3 | 27.85 | -1.86 | 6.E-03 | 0.72 |
| COX6B2 | 10.86 | 2.07 | 5.E-03 | 0.66 |  | KCNJ9 | 7.02 | -1.93 | 7.E-03 | 0.73 |
| LTF | 134.49 | 1.62 | 5.E-03 | 0.66 |  | TNFRSF13B | 7.36 | -3.09 | 7.E-03 | 0.75 |
| MRAP | 6.13 | 2.13 | 5.E-03 | 0.66 |  | GAPDHP70 | 3.57 | -2.34 | 8.E-03 | 0.76 |
| DSCAS | 4.42 | 3.21 | 6.E-03 | 0.69 |  | RN7SL481P | 8.49 | -1.72 | 8.E-03 | 0.76 |
| GJB2 | 93.21 | 1.94 | 6.E-03 | 0.69 |  | PCAT7 | 6.70 | -1.63 | 8.E-03 | 0.78 |
| TBC1D3D | 15.52 | 1.88 | 6.E-03 | 0.72 |  | MARCHF4 | 95.10 | -1.66 | 8.E-03 | 0.78 |
| GARIN5A | 7.79 | 1.68 | 6.E-03 | 0.72 |  | RPL34P24 | 2.69 | -3.29 | 8.E-03 | 0.78 |
| SLC35F3 | 7.28 | 1.89 | 6.E-03 | 0.72 |  | NDST3 | 14.19 | -1.63 | 9.E-03 | 0.79 |
| GRHL2 | 21.73 | 2.14 | 7.E-03 | 0.73 |  | NELL2 | 178.83 | -1.75 | 9.E-03 | 0.79 |
| GAPDHP2 | 4.55 | 2.45 | 7.E-03 | 0.75 |  | RNU6-1189P | 6.41 | -3.41 | 9.E-03 | 0.79 |
| SF3A3P2 | 5.66 | 2.21 | 7.E-03 | 0.75 |  | IGLV3-16 | 6.99 | -4.08 | 9.E-03 | 0.80 |
| ZNF705EP | 8.22 | 2.03 | 8.E-03 | 0.77 |  | ISL1 | 17.49 | -1.77 | 1.E-02 | 0.81 |
| PCGF2 | 78.59 | 3.67 | 8.E-03 | 0.77 |  |  | | | | |
| CALCA | 23.81 | 2.44 | 8.E-03 | 0.78 |  |  |  |  |  |  |
| EGFL8 | 7.52 | 1.90 | 9.E-03 | 0.79 |  |  |  |  |  |  |
| GSX2 | 3.40 | 3.36 | 9.E-03 | 0.80 |  |  |  |  |  |  |
| LY6D | 3.95 | 3.65 | 9.E-03 | 0.80 |  |  |  |  |  |  |

baseMean: the average of the normalized count values, log2FC: log2 of the fold change in gene expression between HD vs non-HD, lfcSE: standard error of the log2 fold change estimate for a gene, stat: the Wald test statistic of DESeq2, pvalue: p-value before adjusted, padj: p-value adjusted for multiple testing using the Benjamini-Hochberg (BH) method. Bold genes indicate significantly upregulated in Figure 1A

**Table S4. Gene Ontology enrichment analysis for upregulated genes**

| ID | Description | GeneRatio | BgRatio | RichFactor | FoldEnrichment | zScore | pvalue | p.adjust | qvalue | geneID | Count |
| --- | --- | --- | --- | --- | --- | --- | --- | --- | --- | --- | --- |
| GO:0050830 | defense response to Gram-positive bacterium | 4/25 | 130/18760 | 0.031 | 23.089 | 9.232 | 2.49E-05 | 0.0182 | 0.0130 | CALCA/CRP/LTF/PLA2G2A | 4 |
| GO:0042742 | defense response to bacterium | 5/25 | 342/18760 | 0.015 | 10.971 | 6.798 | 7.70E-05 | 0.0273 | 0.0194 | CALCA/CRP/LTF/PLA2G2A/WFDC2 | 5 |
| GO:0019732 | antifungal humoral response | 2/25 | 12/18760 | 0.167 | 125.067 | 15.704 | 1.12E-04 | 0.0273 | 0.0194 | CALCA/LTF | 2 |
| GO:0019731 | antibacterial humoral response | 3/25 | 79/18760 | 0.038 | 28.496 | 8.946 | 1.55E-04 | 0.0283 | 0.0202 | CALCA/LTF/WFDC2 | 3 |
| GO:0048705 | skeletal system morphogenesis | 4'25 | 230/18760 | 0.017 | 13.050 | 6.717 | 2.27E-04 | 0.0333 | 0.0237 | GRHL2/LTF/PCGF2/STC1 | 4 |
| GO:0001503 | ossification | 5/25 | 454/18760 | 0.011 | 8.264 | 5.724 | 2.89E-04 | 0.0353 | 0.0252 | CALCA/CHRDL2/LTF/SBNO2/STC1 | 5 |
| GO:0030316 | osteoclast differentiation | 3/25 | 112/18760 | 0.027 | 20.100 | 7.406 | 4.33E-04 | 0.0443 | 0.0315 | CALCA/LTF/SBNO2 | 3 |
| GO:0046697 | decidualization | 2/25 | 25/18760 | 0.080 | 60.032 | 10.789 | 5.02E-04 | 0.0443 | 0.0315 | GJB2/STC1 | 2 |
| GO:0035988 | chondrocyte proliferation | 2/25 | 26/18760 | 0.077 | 57.723 | 10.573 | 5.43E-04 | 0.0443 | 0.0315 | LTF/STC1 | 2 |

**Table S5. Results from Hallmark pathway analysis**

| **pathway** | **log2err** | **ES** | **NES** | **pval** | **padj** |
| --- | --- | --- | --- | --- | --- |
| HALLMARK_MYC_TARGETS_V2 | 0.801 | 0.628 | 2.570 | 8.67E-10 | 3.11E-08 |
| HALLMARK_MYC_TARGETS_V1 | 0.775 | 0.419 | 2.071 | 2.41E-09 | 4.01E-08 |
| HALLMARK_OXIDATIVE_PHOSPHORYLATION | 0.477 | 0.312 | 1.540 | 0.001 | 0.006 |
| HALLMARK_TNFA_SIGNALING_VIA_NFKB | 0.477 | 0.314 | 1.533 | 0.001 | 0.006 |
| HALLMARK_UNFOLDED_PROTEIN_RESPONSE | 0.381 | 0.314 | 1.437 | 0.011 | 0.053 |
| HALLMARK_EPITHELIAL_MESENCHYMAL_TRANSITION | 0.171 | 0.233 | 1.155 | 0.171 | 0.348 |
| HALLMARK_ANGIOGENESIS | 0.125 | 0.313 | 1.131 | 0.260 | 0.464 |
| HALLMARK_DNA_REPAIR | 0.119 | 0.225 | 1.069 | 0.306 | 0.493 |
| HALLMARK_XENOBIOTIC_METABOLISM | 0.110 | 0.211 | 1.021 | 0.386 | 0.555 |
| HALLMARK_REACTIVE_OXYGEN_SPECIES_PATHWAY | 0.084 | 0.248 | 0.956 | 0.513 | 0.674 |
| HALLMARK_INFLAMMATORY_RESPONSE | 0.083 | 0.192 | 0.945 | 0.592 | 0.674 |
| HALLMARK_FATTY_ACID_METABOLISM | 0.077 | 0.196 | 0.929 | 0.620 | 0.674 |
| HALLMARK_APICAL_SURFACE | 0.075 | 0.243 | 0.909 | 0.608 | 0.674 |
| HALLMARK_E2F_TARGETS | 0.071 | 0.182 | 0.898 | 0.755 | 0.771 |
| HALLMARK_CHOLESTEROL_HOMEOSTASIS | 0.041 | -0.160 | -0.647 | 0.984 | 0.984 |
| HALLMARK_SPERMATOGENESIS | 0.054 | -0.201 | -0.867 | 0.744 | 0.771 |
| HALLMARK_UV_RESPONSE_UP | 0.055 | -0.196 | -0.897 | 0.696 | 0.740 |
| HALLMARK_PANCREAS_BETA_CELLS | 0.068 | -0.287 | -0.908 | 0.584 | 0.674 |
| HALLMARK_MTORC1_SIGNALING | 0.060 | -0.196 | -0.943 | 0.603 | 0.674 |
| HALLMARK_IL6_JAK_STAT3_SIGNALING | 0.068 | -0.232 | -0.944 | 0.558 | 0.674 |
| HALLMARK_NOTCH_SIGNALING | 0.070 | -0.279 | -0.950 | 0.544 | 0.674 |
| HALLMARK_PI3K_AKT_MTOR_SIGNALING | 0.070 | -0.226 | -0.969 | 0.530 | 0.674 |
| HALLMARK_G2M_CHECKPOINT | 0.077 | -0.212 | -1.017 | 0.425 | 0.574 |
| HALLMARK_IL2_STAT5_SIGNALING | 0.080 | -0.215 | -1.031 | 0.400 | 0.555 |
| HALLMARK_ESTROGEN_RESPONSE_EARLY | 0.081 | -0.216 | -1.032 | 0.392 | 0.555 |
| HALLMARK_COAGULATION | 0.090 | -0.240 | -1.041 | 0.367 | 0.555 |
| HALLMARK_INTERFERON_ALPHA_RESPONSE | 0.094 | -0.250 | -1.061 | 0.333 | 0.520 |
| HALLMARK_HYPOXIA | 0.097 | -0.227 | -1.080 | 0.294 | 0.490 |
| HALLMARK_GLYCOLYSIS | 0.098 | -0.227 | -1.084 | 0.287 | 0.490 |
| HALLMARK_ADIPOGENESIS | 0.126 | -0.238 | -1.138 | 0.188 | 0.348 |
| HALLMARK_UV_RESPONSE_DN | 0.134 | -0.250 | -1.143 | 0.181 | 0.348 |
| HALLMARK_ALLOGRAFT_REJECTION | 0.128 | -0.244 | -1.151 | 0.184 | 0.348 |
| HALLMARK_APICAL_JUNCTION | 0.163 | -0.251 | -1.193 | 0.118 | 0.311 |
| HALLMARK_PEROXISOME | 0.145 | -0.279 | -1.193 | 0.160 | 0.348 |
| HALLMARK_BILE_ACID_METABOLISM | 0.146 | -0.278 | -1.196 | 0.161 | 0.348 |
| HALLMARK_INTERFERON_GAMMA_RESPONSE | 0.168 | -0.251 | -1.197 | 0.111 | 0.309 |
| HALLMARK_ESTROGEN_RESPONSE_LATE | 0.180 | -0.257 | -1.218 | 0.099 | 0.298 |
| HALLMARK_MYOGENESIS | 0.185 | -0.258 | -1.222 | 0.095 | 0.298 |
| HALLMARK_ANDROGEN_RESPONSE | 0.161 | -0.286 | -1.225 | 0.133 | 0.332 |
| HALLMARK_TGF_BETA_SIGNALING | 0.151 | -0.323 | -1.236 | 0.152 | 0.348 |
| HALLMARK_KRAS_SIGNALING_UP | 0.217 | -0.263 | -1.248 | 0.069 | 0.248 |
| HALLMARK_COMPLEMENT | 0.322 | -0.275 | -1.298 | 0.037 | 0.154 |
| HALLMARK_APOPTOSIS | 0.288 | -0.288 | -1.332 | 0.042 | 0.163 |
| HALLMARK_WNT_BETA_CATENIN_SIGNALING | 0.186 | -0.370 | -1.333 | 0.101 | 0.298 |
| HALLMARK_PROTEIN_SECRETION | 0.352 | -0.326 | -1.396 | 0.021 | 0.097 |
| HALLMARK_KRAS_SIGNALING_DN | 0.455 | -0.324 | -1.501 | 0.002 | 0.012 |
| HALLMARK_HEME_METABOLISM | 0.498 | -0.342 | -1.625 | 0.000 | 0.003 |
| HALLMARK_HEDGEHOG_SIGNALING | 0.407 | -0.492 | -1.712 | 0.004 | 0.024 |
| HALLMARK_P53_PATHWAY | 0.593 | -0.373 | -1.782 | 1.42E-05 | 0.000178 |
| HALLMARK_MITOTIC_SPINDLE | 0.788 | -0.434 | -2.082 | 1.24E-09 | 3.11E-08 |

**Table S6. Cibersortx results**

| Mixture | group | B cells naive | B cells memory | Plasma cells | T cells CD8 | T cells CD4 naive | T cells CD4 memory resting | T cells CD4 memory activated | T cells follicular helper | T cells regulatory (Tregs) | T cells gamma delta | NK cells resting | NK cells activated |
| --- | --- | --- | --- | --- | --- | --- | --- | --- | --- | --- | --- | --- | --- |
| AS1 | non-HD | 0.1017754 | 0 | 0.081087655 | 0 | 0 | 0.180320539 | 0 | 0.014610663 | 0 | 0 | 0.01561773 | 0.007136143 |
| AS2 | non-HD | 0.052277839 | 0 | 0.049257845 | 0.00458009 | 0 | 0.132241549 | 0.014675845 | 0 | 0.019075306 | 0 | 0.032648152 | 0 |
| AS4 | non-HD | 0.141959567 | 0 | 0 | 0.00241504 | 0 | 0.192877422 | 0 | 0 | 0.011979788 | 0 | 0.034848303 | 0.002951407 |
| AS5 | non-HD | 0.099676012 | 0 | 0.014523824 | 0.00653385 | 0 | 0.217496243 | 0 | 0 | 0.008909694 | 0 | 0.004108489 | 0.018743316 |
| ASHD1 | HD | 0.10268965 | 0 | 0.101166194 | 0 | 0 | 0.122425145 | 0.017376269 | 0 | 0 | 0 | 0.01968248 | 0.001522788 |
| ASHD2 | HD | 0.082253377 | 0 | 0.002812392 | 0.00812246 | 0 | 0.150581934 | 0 | 0.003420547 | 0.012387876 | 0 | 0.029287403 | 0.008765353 |
| ASHD3 | HD | 0.064331458 | 0 | 0.006245803 | 0.00673166 | 0 | 0.262941651 | 1.85E-04 | 0 | 3.19E-04 | 0 | 0.034880239 | 0.001802561 |
| ASHD4 | HD | 0.053612486 | 0 | 0.036623067 | 0.0062272 | 0 | 0.175399662 | 0.005215861 | 0.009473486 | 0.006786671 | 0 | 0.011625559 | 0.003233702 |
| ASHD5 | HD | 0.078249095 | 0 | 0.013947969 | 0 | 0 | 0.120079075 | 0.001416156 | 0 | 0 | 0 | 0.024368005 | 0 |

| Mixture | group | Monocytes | Macrophages M0 | Macrophages M1 | Macrophages M2 | Dendritic cells resting | Dendritic cells activated | Mast cells resting | Mast cells activated |
| --- | --- | --- | --- | --- | --- | --- | --- | --- | --- |
| AS1 | non-HD | 0.01497822 | 0.175506857 | 0.028903251 | 0.290889577 | 0 | 0 | 0.087709064 | 0 |
| AS2 | non-HD | 0.05299214 | 0.158922137 | 0.02361275 | 0.385332404 | 0 | 0 | 0.062478577 | 0 |
| AS4 | non-HD | 0.06498149 | 0 | 0.033112479 | 0.41992704 | 0 | 0 | 0.091161074 | 0 |
| AS5 | non-HD | 0.03364636 | 0.007841549 | 0.031220759 | 0.435848428 | 0 | 0 | 0.075609114 | 0 |
| ASHD1 | HD | 0.15874148 | 0.081979097 | 0.031782653 | 0.312836553 | 0 | 0 | 0.026188231 | 0 |
| ASHD2 | HD | 0.08475141 | 0.032155379 | 0.035450836 | 0.460236018 | 0 | 0 | 0.069569682 | 0 |
| ASHD3 | HD | 0.0172059 | 0.114554711 | 0.054648204 | 0.344990765 | 0 | 0 | 0.068657611 | 0 |
| ASHD4 | HD | 5.57E-04 | 0.244482445 | 0.032404914 | 0.33220774 | 0 | 0 | 0.079252779 | 0 |
| ASHD5 | HD | 1.93E-05 | 0.261407201 | 0.018777695 | 0.37295161 | 0 | 0 | 0.105550068 | 0 |

| Mixture | group | Eosinophils | Neutrophils | Macrophages_total | Mast_cells | T cells CD4 memory | Dendritic cells | NK cells | P-value | Correlation | RMSE |
| --- | --- | --- | --- | --- | --- | --- | --- | --- | --- | --- | --- |
| AS1 | non-HD | 0 | 0.0014649 | 0.495299685 | 0.0877091 | 0.180320539 | 0 | 0.02275 | 0 | 0.7707758 | 0.724010415 |
| AS2 | non-HD | 0 | 0.0119054 | 0.567867292 | 0.0624786 | 0.146917394 | 0 | 0.03265 | 0 | 0.8182372 | 0.67190655 |
| AS4 | non-HD | 0 | 0.0037864 | 0.453039519 | 0.0911611 | 0.192877422 | 0 | 0.0378 | 0 | 0.6521792 | 0.788421669 |
| AS5 | non-HD | 0 | 0.0435735 | 0.474910735 | 0.0756091 | 0.217496243 | 0 | 0.02285 | 0 | 0.6481824 | 0.788562297 |
| ASHD1 | HD | 0 | 0.0236095 | 0.426598303 | 0.0261882 | 0.139801414 | 0 | 0.02121 | 0 | 0.4900401 | 0.873511026 |
| ASHD2 | HD | 0 | 0.0100343 | 0.527842233 | 0.0695697 | 0.150581934 | 0 | 0.03805 | 0 | 0.6879562 | 0.758449105 |
| ASHD3 | HD | 0 | 0.0099215 | 0.514193681 | 0.0686576 | 0.263126366 | 0 | 0.03668 | 0 | 0.7496083 | 0.728267646 |
| ASHD4 | HD | 0 | 0.0028976 | 0.609095099 | 0.0792528 | 0.180615522 | 0 | 0.01486 | 0 | 0.8195004 | 0.659719985 |
| ASHD5 | HD | 0 | 0.0032338 | 0.653136507 | 0.1055501 | 0.121495231 | 0 | 0.02437 | 0 | 0.7533715 | 0.695055024 |
